# Complementary school garden, nutrition, water, sanitation and hygiene interventions to improve children’s nutrition and health status in Burkina Faso and Nepal: a study protocol

**DOI:** 10.1186/s12889-016-2910-7

**Published:** 2016-03-09

**Authors:** Séverine Erismann, Akina Shrestha, Serge Diagbouga, Astrid Knoblauch, Jana Gerold, Ramona Herz, Subodh Sharma, Christian Schindler, Peter Odermatt, Axel Drescher, Ray-yu Yang, Jürg Utzinger, Guéladio Cissé

**Affiliations:** Swiss Tropical and Public Health Institute, P.O. Box, CH-4002, Basel, Switzerland; University of Basel, P.O. Box, CH-4003, Basel, Switzerland; Kathmandu University, P.O. Box 6250, 45200 Dhulikhel, Nepal; Institut de Recherches en Sciences de la Santé, P.O. Box 7192, Ouagadougou, 03 Burkina Faso; University of Freiburg, Friedrichstr. 39, D-79098 Freiburg im Breisgau, Germany; AVRDC - The World Vegetable Center, P.O. Box 42, 74151 Shanhua, Taiwan

**Keywords:** Burkina Faso, Malnutrition, Nepal, Parasitic infections, School-aged children, Study protocol, Water, Sanitation and hygiene (WASH)

## Abstract

**Background:**

Malnutrition and intestinal parasitic infections are common among children in Burkina Faso and Nepal. However, specific health-related data in school-aged children in these two countries are scarce. In the frame of a larger multi-stakeholder project entitled “Vegetables go to School: Improving Nutrition through Agricultural Diversification” (VgtS), a study has been designed with the objectives to: (i) describe schoolchildren’s health status in Burkina Faso and Nepal; and to (ii) provide an evidence-base for programme decisions on the relevance of complementary school garden, nutrition, water, sanitation and hygiene (WASH) interventions.

**Methods/Design:**

The studies will be conducted in the Centre Ouest and the Plateau Central regions of Burkina Faso and the Dolakha and Ramechhap districts of Nepal. Data will be collected and combined at the level of schools, children and their households. A range of indicators will be used to examine nutritional status, intestinal parasitic infections and WASH conditions in 24 schools among 1144 children aged 8–14 years at baseline and a 1-year follow-up. The studies are designed as cluster randomised trials and the schools will be assigned to two core study arms: (i) the ‘complementary school garden, nutrition and WASH intervention’ arm; and the (ii) ‘control’ arm with no interventions. Children will be subjected to parasitological examinations using stool and urine samples and to quality-controlled anthropometric and haemoglobin measurements. Drinking water will be assessed for contamination with coliform bacteria and faecal streptococci. A questionnaire survey on nutritional and health knowledge, attitudes and practices (KAP) will be administered to children and their caregivers, also assessing socioeconomic, food-security and WASH conditions at household level. Focus group and key-informant interviews on children’s nutrition and hygiene perceptions and behaviours will be conducted with their caregivers and school personnel.

**Discussion:**

The studies will contribute to fill a data gap on school-aged children in Burkina Faso and Nepal. The data collected will also serve to inform the design of school-based interventions and will contribute to deepen the understanding of potential effects of these interventions to improve schoolchildren’s health in resource-constrained settings. Key findings will be used to provide guidance for the implementation of health policies at the school level in Burkina Faso and Nepal.

**Trial registration:**

ISRCTN30840 (date assigned: 17 July 2015)

## Background

Malnutrition, intestinal parasitic infections and diarrhoeal diseases are common public health problems in children in low- and middle-income countries (LMIC) [1–8]. In many countries, Demographic and Health Surveys (DHS) and national nutrition surveillance systems have been measuring height and weight of children below the age of 5 years, starting in the early 1990s. However, there is a paucity of anthropometric data for school-aged children (5–14 years) [9–11]. Additionally, there are currently no estimates neither for school-aged children, nor the entire population, on the global burden of diseases from polyparasitism of intestinal parasitic infections caused by helminths and intestinal protozoa [7]. Data on the burden of disease caused by intestinal protozoa is scarce, partially explained by the lack of diagnosis at the periphery [12–15]. Although no estimates on the burden of diseases caused by helminth infections for school-aged children exist, an estimate for the burden of disease of sub-groups of helminth infections is available (e.g. schistosomiasis and soil-transmitted helminthiasis) [4, 7, 16]. Estimates from the Global Atlas of Helminth Infection (GAHI; http://www.thiswormyworld.org/) showed that, in 2010, 1.01 billion school-aged children lived in areas where prevalence of any soil-transmitted helminth (STH) was above 20 % [7]. Furthermore, in 2013, diarrhoeal diseases were responsible for an estimated 7 % of deaths in school-aged children in LMICs, with more than 96 % attributable to unsafe water, inadequate sanitation and hygiene (WASH) [4, 5].

Burkina Faso and Nepal are both low-income countries that face an array of similar health challenges. Whilst health data among under 5-year-old children, such as nutritional indicators, anaemia or *Plasmodium* prevalence, are collected during national surveys, statistics on school-aged children in these two countries are typically unavailable [17, 18]. Malnutrition, anaemia and diarrhoeal diseases are highly prevalent in under 5-year-old children. Indeed, according to the 2010 and 2011 DHS in Burkina Faso and Nepal, respectively, 35 % and 41 % of children were stunted; almost 15 % of children in both countries reported diarrhoea 2 weeks prior to a DHS; and 88 % of the children in Burkina Faso and 46 % in Nepal were anaemic [17, 18]. Both countries also face considerable ill-health due to inadequate WASH conditions. For example, according to data from the 2013 Global Burden of Disease Study (GBD) and the World Health Organization (WHO)/United Nations Children's Fund (UNICEF) ‘Joint Monitoring Programme for Water Supply and Sanitation’, 7 % and 8 % of deaths in children aged 8–14 years in Burkina Faso and Nepal, respectively, were caused by diarrhoeal diseases, with over 96 % in both countries attributed to inadequate WASH conditions as primary risk factor [4, 19]. Table 1 provides an overview of selected health and WASH indicators in Burkina Faso and Nepal for the years 2010 to 2013.

**Table 1:** Overview of health and WASH indicators of Burkina Faso and Nepal: (a) Mortality rate among children aged 5 to 14 years old; (b) Disability-adjusted life year (DALYs) as indicator of morbidity among children aged 5 to 14 years old

Indicator	Burkina Faso	Nepal
Health	*DHS 2010*	*DHS 2011*
Stunting (<5 years)	35 %	41 %
Wasting (<5 years)	16 %	11 %
Underweight (<5 years)	26 %	29 %
Diarrhoea (<5 years)	15 %	14 %
Anaemia (<5 years)	88 %	46 %
Mortality (a) and morbidity [DALYs] (b)	*GBD 2013*	*GBD 2013*
Diarrhoeal diseases (5 to 14 years old)	7 % (a), 5 % (b)	8 % (a), 4 % (b)
Iron-deficiency anaemia (5 to 14 years old)	1 % (a), 6 % (b)	1 % (a), 15 % (b)
Intestinal infectious diseases (5 to 14 years old)	4 % (a), 2 % (b)	10 % (a), 4 % (b)
Water, sanitation and hygiene (WASH)	*DHS 2010 (a) and WHO Progress Report on Drinking-Water and Sanitation 2012 (b)*	*DHS 2011 (a) and WHO Progress Report on Drinking-Water and Sanitation 2012 (b)*
Improved latrines	15 % (a), 19 % (b)	38 % (a), 37 % (b)
Non-improved latrines	6 % (a), 17 % (b)	43 % (a), 6 % (b)
Open defaecation (bush/field, no latrines)	62 % (a), 57 % (b)	36 % (a), 40 % (b)
Soap and water for hand washing available	14 % (a)	48 % (a)

(a) Mortality rate among children aged 5 to 14 years old

(b) Disability-adjusted life year (DALYs) as indicator of morbidity among children aged 5 to 14 years old

Malnutrition, intestinal parasitic infections and inadequate WASH conditions are intricately linked. First, inadequate WASH conditions are important risk factors for both, malnutrition and intestinal parasitic infections [2, 4, 15, 20, 21]. The pathogenic agents associated with poor WASH conditions are viral pathogens, bacterial pathogens, protozoan cysts or oocysts and helminth eggs found in faeces and transmitted through the faecal-oral pathway and can lead to diarrhoea and undernutrition, whereby exposure to one increases vulnerability to the other [22–27]. Second, malnutrition can render a child more susceptible to infection. An inadequate dietary intake leads to weight loss, weakened immunity, invasion by pathogens and mucosal damage, and impaired growth and development in children [28–30]. Third, parasitic infections also contribute to growth stunting by causing a decline in food intake (loss of appetite), diarrhoea, malabsorption and/or an increase in nutrient wastage for the immune response, all of which lead to nutrient losses and further damage to the defence mechanisms, causing a vicious cycle [28–30]. Moreover, it is well documented that infections with intestinal parasites may cause internal bleeding, leading to a loss of iron and anaemia [31, 32]. Intestinal parasitic infections can go unnoticed for years due to delayed onset of symptoms, which can exacerbate the effects on malnutrition, and hence compromise the development of their cognitive abilities in their formative years [30]. It is therefore crucial to consider the strong interlinkages of malnutrition, parasitic infections, diarrhoeal diseases and WASH for preventive actions and sustainable programmes.

### “Vegetables go to School: Improving Nutrition through Agricultural Diversification”

A multi-country and multi-stakeholder project entitled “Vegetables go to School: Improving Nutrition through Agricultural Diversification” (VgtS in short) was developed and is funded by the Swiss Agency for Development and Cooperation (SDC) to address schoolchildren’s nutrition in an interdisciplinary approach through the implementation of school vegetable gardens and other school-based health, nutrition and environmental interventions. The VgtS project was launched in 2012 in six target countries (Bhutan, Burkina Faso, Indonesia, Nepal, the Philippines and Tanzania) and is implemented by country teams composed of members of different ministries, (i.e. education, agriculture and health), in collaboration with the World Vegetable Center (AVRDC; headquartered in Taïwan), the University of Freiburg in Germany and the Swiss Tropical and Public Health Institute (Swiss TPH) in Switzerland as academic partners.

The objectives of the VgtS project are threefold: (i) to encourage agricultural production at the unit of the school and to increase the availability and access to a wide diversity of vegetables in order to favour a balanced and nutritious diet; (ii) to link the school garden to an educational programme that covers basic topics of agriculture, nutrition and WASH (overall project approach in all the countries); and (iii) to link the school garden programme to complementary nutrition and WASH interventions. In this context, the VgtS project embeds two intervention studies in Burkina Faso and Nepal, which include intervention schools benefitting from a complementary school garden, nutrition and WASH intervention package and control schools without any intervention. Here, we present the research protocol for the studies in Burkina Faso and Nepal.

## Methods/Design

### Goal

The overarching goal of the studies within the frame of the VgtS project in Burkina Faso and Nepal is to address the current data gap on schoolchildren (aged 8–14 years) and to assess the effects of complementary school garden, nutrition and WASH interventions on schoolchildren’s health status, as assessed by a baseline and a 1-year follow-up survey through a range of previously identified nutrition, WASH and health indicators (Table 2).

**Table 2:** Selected indicators for the two studies in Burkina Faso and Nepal

Objective	Indicator	Methods and tools	Survey module
*Individual level of child*			
To assess schoolchildren’s nutritional status at baseline and follow-up	Nutritional status (BMIZ, HAZ, WAZ and clinical signs of malnutrition)	Digital scale, height measuring board and clinical examination	Nutritional survey (module 1)
To assess the prevalence of anaemia in schoolchildren at baseline and follow-up	Anaemia based on haemoglobin levels < 11.5 g/dl for children aged 7 to 11 years and < 12 g/dl for those aged 12 to 14 years	HemoCue Hb 201^+^	Nutritional survey (module 1)
To assess the prevalence of intestinal parasitic infections in schoolchildren at baseline and follow-up	Presence and intensity of intestinal and urinary parasitic infections	Kato-Katz and formalin-ether concentration method for stool samples and centrifugation method for urine samples	Parasitological survey (module 2)
To assess schoolchildren’s nutrition and health knowledge, attitudes and practices (KAP) at baseline and follow-up	KAP related to nutrition and health	Questionnaire survey with schoolchildren Focus group discussions with schoolchildren In-depth interviews with school directors and teachers	Children’s health KAP (module 3)
*Environmental indicators*			
To assess drinking water quality of children’s drinking water recipients at baseline and follow-up	Presence of thermotolerant coliform bacteria and faecal streptococci	Portable DelAgua field kit and RAPID E. COLI 2 AGAR (coliform bacteria, *Escherichia coli*) and Bile Esculine Azide AGAR (faecal streptococci) tests	Water quality testing (module 4)
*Household level*			
* Demographic and socioeconomic data*			
To assess basic household socio-demographic and economic characteristics at baseline and follow-up	Caregiver’s age, educational level, occupational status, assets, food security	Household questionnaire	Household questionnaire survey (module 5)
* Household nutrition and health -related knowledge, attitudes and practices data*			
To assess caregivers’ nutrition and health knowledge, attitudes and practices at baseline and follow-up	Caregiver’s knowledge, attitudes and practices related to nutrition and health	Household questionnaire Focus group discussions with schoolchildren’s caregivers	Household questionnaire survey (module 5) Caregivers’ health knowledge, attitudes and practices (module 6)
* Socio-environmental conditions data*			
To assess household WASH conditions at baseline and follow-up	Drinking water source and distance to it, water storage, improved/non-improved latrine, location of kitchen, available hand washing facilities and soap, presence of domestic animals	Household living condition and information related to hygiene Direct observation	Household questionnaire survey (module 5)
* Environmental indicators*			
To assess drinking water quality at schoolchildren’s households at baseline and follow-up	Presence of thermotolerant coliform bacteria and faecal streptococci	Portable DelAgua field kit and RAPID E. COLI 2 AGAR (coliform bacteria, *Escherichia coli*) and Bile Esculine Azide AGAR (faecal streptococci) tests	Water quality testing (module 4)
*School and community level*			
* Socio-environmental conditions data*			
To assess the WASH conditions at schools at baseline and follow-up	Available drinking water, available improved/non-improved toilet/latrine, available hand washing facilities and soap	In-depth interviews with school directors and teachers Direct observation	WASH survey (module 7)
* Environmental indicators*			
To assess drinking water quality at schools and community sources at baseline and follow-up	Presence of thermotolerant coliform bacteria and faecal streptococci	Portable DelAgua field kit and RAPID E. COLI 2 AGAR (coliform bacteria, *Escherichia coli*) and Bile Esculine Azide AGAR (faecal streptococci) tests	Water quality testing (module 4)

### Study sites and school selection

The studies will be conducted in Burkina Faso and Nepal. The study sites are located within the VgtS project sites, which were selected by the local VgtS country teams, following a set of criteria: (i) accessibility from the capital; (ii) availability of land for the school garden and continuous access to water at schools; (iii) coeducation of boys and girls in public schools; and (iv) willingness of the school principals and the community to participate.

In both countries, the study will be implemented in two different regions. In Burkina Faso, these are the Centre Ouest and the Plateau Central regions, both located in proximity to the capital Ouagadougou (30–180 km). The study sites in Nepal are the Dolakha and Ramechhap districts in the eastern part of the country, both located in proximity of the district headquarters Charikot (133 km) and Manthali (131 km), respectively.

**Fig. 1:**
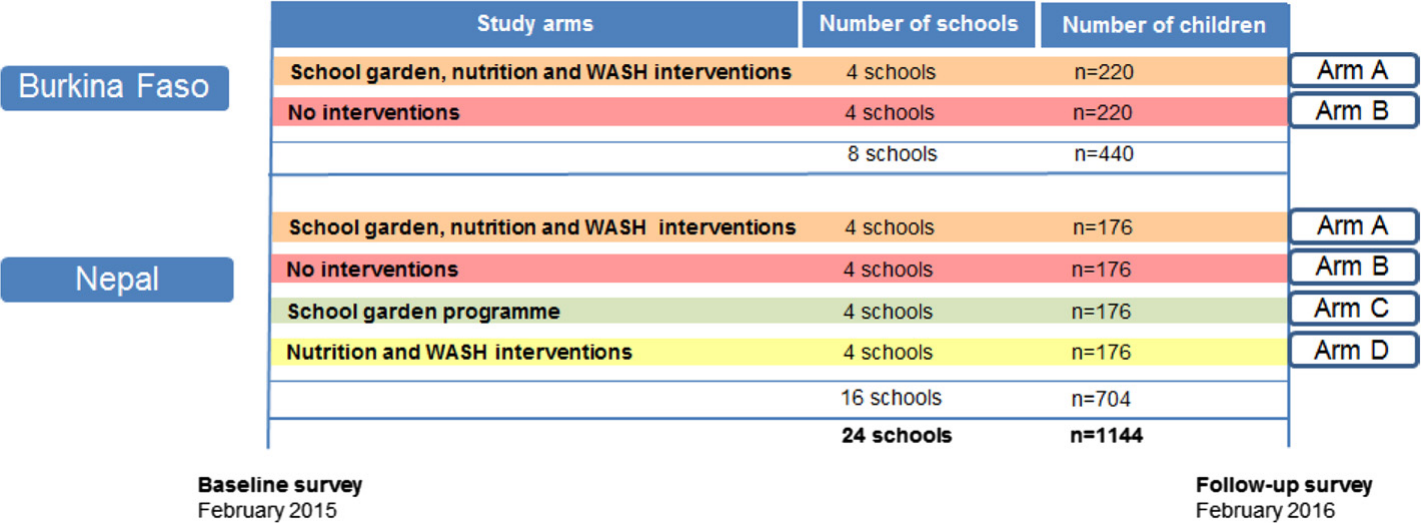
Study design for Burkina Faso and Nepal

The selection of the schools participating in the two studies is based on a three-stage sampling procedure of schools within the overall VgtS project sites. In a first step, about 100 schools fulfilling the aforementioned eligibility criteria were selected. In a second step, from these 100 schools, a sample of 30 schools were randomly chosen to be included in the VgtS school garden implementation and were randomly allocated to three groups, which receive the school vegetable garden interventions in 2014, 2015 and 2016, respectively. In a third step, out of the 30 VgtS project schools, a total of eight schools in Burkina Faso and 16 schools in Nepal were randomly selected to accommodate the sampling needs of the two complementary and slightly different study designs of Burkina Faso and Nepal (Fig. 1).

### Study design

The two studies in Burkina Faso and Nepal are designed as cluster randomised trials with an equal number of schools randomly allocated to two core study arms (A, B) and with a cohort of children followed in two consecutive surveys, at baseline and 1-year follow-up. Two additional study arms are included in Nepal (C, D). The four study arms are designed as follows:

- arm A: school garden programme and complementary nutrition and WASH interventions;
- arm B: no interventions, i.e. controls;
- arm C: school garden programme without nutrition and WASH interventions; and
- arm D: nutrition and WASH interventions without the school garden programme.

Each arm comprises four schools. Figure 1 shows the study design with the different study arms for Burkina Faso and Nepal. In both countries, schools of arm A will receive the complementary school garden, nutrition and WASH intervention package starting in March 2015. In Nepal, the interventions from arms C and D will be implemented over the same period. In both countries, the control schools of arm B will benefit from the school garden intervention in the year following the interventions.

### Sample size

Two separate sample size calculations were conducted for the two study designs of Burkina Faso and Nepal. For the sample size calculation of the study in Burkina Faso, the prevalence of intestinal parasitic infection in children aged 8–14 years was selected as the primary outcome in the comparison between high- and low-risk of intestinal parasitic infection in children. The power calculation was based on the assumption of:

- an average intestinal protozoa and helminth infection rate across schools of 40 % [33];
- a coefficient of variation of 10 % across schools; and
- a proportion of high risk children of 25 %.

A Monte Carlo simulation with 5000 iterations shows that a total of 400 children from eight schools (i.e. 50 children per school) would provide 85 % power for detecting a significant difference in infection rates between high- and low-risk children at the usual level of 5 % under these assumptions and for a true odds ratio of 2. Recruitment will be increased by 10 % to account for drop-outs or non-participation, which leads to an optimal sample size of at least 440 children.

The sample size calculation for the study in Nepal was also based on the prevalence of intestinal parasitic infections in children aged 8–14 years as the primary outcome. The power calculation was based on the assumption of:

- the prevalence rate of intestinal protozoa and helminth infection is 30 % [34] and this rate will remain in steady state in the absence of any intervention;
- probability of new intestinal protozoa and helminth infection at the 1-year follow-up will be reduced by at least 10 % through the implementation of the complementary nutrition and WASH intervention package; and
- a coefficient of variation of 10 % across schools.

A Monte Carlo simulation with 5000 iterations shows that a total of 640 schoolchildren from 16 schools (i.e. 40 children per school) would provide 80 % of power for detecting a significant difference in infection rates between the four study arms. Recruitment will be increased by 10 % accounting for drop-outs and non-participants, which leads to an optimal sample size of at least 704 children.

### Eligibility and selection criteria of study participants

In both Burkina Faso and Nepal, children enrolled in school are eligible to participate in the baseline survey if they are between 8 and 14 years old, have signed a written informed consent by their parents, guardians or teachers, and themselves assented orally.

### Data collection procedures

Four weeks prior to the study, district and village authorities, school directors and children’s parents/guardians will be informed about the forthcoming survey activities by the local survey team. They will be re-informed about the purpose and procedures of the study shortly before the start of the survey activities. Written informed consent (signed or fingerprint) will be obtained from children’s parents or legal guardians, whilst children will assent orally. It will be emphasised that participation is voluntary and that children and parents/guardians can withdraw anytime without further obligation.

In each school, a random selection of children aged 8–14 years will be enrolled until at least 55 in Burkina Faso and 44 in Nepal are reached. Moreover, at the follow-up survey, the same children will be re-assessed, who by then will be 9–15 years old. Each child will be attributed a unique identification code (ID) for the different assessments at the onset of the study. A separate household ID connected to the schoolchild’s personal ID will be given to children’s households in order to link children’s clinical data and nutritional and health knowledge, attitudes and practices (KAP) with the household characteristics. Children will thereafter be invited to provide stool and urine samples, to take anthropometric and haemoglobin (Hb) measurements and to participate in the KAP survey. In Burkina Faso, stool and urine samples will be collected on two consecutive days. In Nepal, a single stool sample will be collected, while urine samples will not be collected as urogenital schistosomiasis is not endemic. Infected, anaemic or undernourished children in all schools will be subjected to clinical, parasitological and nutritional examinations, and will be treated according to national policies.

After these assessments with children at the schools, the same enumerators in Burkina Faso will visit children’s households and will invite children’s caregivers to respond to a household questionnaire during the two survey days. In Nepal, due to the scattered locations and geographical constraints, additional enumerators will visit the children’s households during the same survey period. In both countries, trained and experienced enumerators will conduct the questionnaire surveys in local languages.

### Collection of stool and urine samples

The sampled children at the schools will be asked to provide a fresh mid-morning, post-exercise stool sample. The stool samples will be processed and analysed each day (at mid-day the latest) by experienced laboratory technicians and medical microbiologists as follows: first, stool samples will be visually examined for macroscopic appearance of adult worms, also checking the stool consistency and the presence of blood and mucus. Second, a single Kato-Katz thick smear, using 41.7 mg templates, will be prepared on a slide and examined under a microscope for the presence of eggs of *Schistosoma mansoni*, hookworm, *Ascaris lumbricoides, Trichuris trichiura* and *Hymenolepis nana*, adhering to standard operating procedures [35, 36].

Third, a formalin-ether concentration technique will be used to enhance sensitivity for the diagnosis of helminths and to detect intestinal protozoa (*Blastocystis hominis*, *Chilomastix mesnili*, *Endolimax nana*, *Entamoeba coli*, *Entamoeba histolytica*/*Entamoeba dispar*, *Entamoeba hartmanni*, *Giardia intestinalis* and *Iodamoeba bütschlii*) [37]. Approximately 1–2 g of stool will be placed in 15 ml Falcon tubes filled with 10 ml of 5 % formalin and will be examined by experienced laboratory technicians for the presence of helminths and intestinal protozoa, using an ether-concentration technique, adhering to an SOP [38]. Additionally, in Nepal, 20 mg of stool will be prepared on a single slide with the saline wet mount concentration for the microscopic detection of the same intestinal protozoa and helminths, according to SOPs [39, 40]. Furthermore, the intensity of infection will be calculated as the number of eggs per 1 g of stool (EPG) and categorised according to the WHO standard classification [41].

In Burkina Faso, children will also be asked to provide fresh, mid-morning and post-exercise urine samples, collected at the same time as the stool samples. Urine samples will be analysed for microhaematuria (biochemical marker and proxy for *Schistosoma haematobium*), using reagent strips (Hemastix; Siemens Healthcare Diagnostics GmbH; Eschborn, Germany) [42], and for the presence and number of *S. haematobium* eggs in 10 ml of urine using a urine centrifugation technique and microscopy [43]. *S. haematobium* infection will be grouped into light (< 50 eggs/10 ml of urine) and heavy (≥ 50 eggs/10 ml of urine) [44].

In order to achieve a higher sensitivity in diagnostics, in selected schoolchildren stool and urine samples will be obtained on two consecutive days in Burkina Faso [45, 46]. For quality control, 10 % of all processed stool samples will be re-read under a microscope by independent laboratories [47]. Slides identified with discrepant results will be re-examined by the Institut de Recherches en Sciences de la Santé (IRSS) laboratory and Kirnetar Health Centre team until agreement has been reached.

### Collection of anthropometric indicators and measuring Hb levels

Selected schoolchildren will be subjected to anthropometric measurements according to SOPs, as described by WHO, using a digital scale and a height measuring board with a precision of 0.1 kg and 0.1 cm, respectively [48]. Individual z-score will be computed using the new WHO growth reference values for children and adolescents [49]. The nutritional status of schoolchildren will be classified as follows: a z-score within the interval of −3 standard deviation (SD) < z < −2 SD will be used to classify body-mass-index-for-age (BMIZ, thinness), height-for-age (HAZ, stunting) and weight-for-age (WAZ, wasting) as moderate undernutrition, and a z-score <−3 SD to define severe undernutrition. WAZ will only be used for children aged 8–10 years as reference data are not available beyond the age of 10 years [49]. Overweight will be classified as BMIZ >1.0 SD and obesity as BMIZ >2.0 SD [50].

The Hb level will be measured to determine anaemia prevalence. A finger-prick capillary blood sample will be taken, and Hb concentration measured using a HemoCue® 201+ testing device (HemoCue Hb 201+ System; HemoCue AB, Ängelholm, Sweden). Age-specific criteria will be used to identify anaemic children: Hb <11.5 g/dl for children aged 8–11 years and Hb <12 g/dl for children aged 12–14 years [51].

Additionally in Nepal, trained health care professionals will conduct clinical examinations for detecting clinical signs of nutritional deficiencies (e.g. dermatitis, bitot’s spot, dry and infected cornea, oedema, enlargement of liver, loss of peripheral sensation, angular stomatitis, pale conjunctiva, red inflamed tongue, swelling of the thyroid gland and bowed legs) [52].

### Drinking water quality assessment

In Burkina Faso, drinking water samples will be collected in sterile 250 ml bottles at the selected schools and community sources, children’s households and from their drinking water recipients to assess drinking water quality at source and point of use. Water samples will be randomly taken in 20 % of participating children’s households and in five community sources per study site (always including the school source). Water samples from children’s drinking water recipients brought to school will be randomly collected in 30 % of the children. Before analysis, the water samples collected will be preserved in cool boxes at 4 °C, and transferred to a nearby laboratory. The water samples will be analysed by membrane filtration for the presence/absence (PA) of thermotolerant faecal coliforms (TTC) as colony forming units per 100 ml of water (CFU/100 ml). Furthermore, *E. coli* and faecal streptococci as indicators for faecal contamination will be assessed by the use of the RAPID E. COLI 2 AGAR (coliform bacteria and *E. coli*; Bio-Rad Laboratories, Hercules, USA) and the Bile Esculine Azide AGAR (faecal streptococci; Bio-Rad Laboratories, Hercules, USA) tests according to WHO drinking water standards [53].

In Nepal, drinking water samples will be collected in 250 ml sterile bottles from the school drinking water source and children’s drinking water recipients, household and community water sources. Water samples will be collected at every school (*n*=16) and every child’s household (*n*=440). For the community sources, one water sample per study site will be taken from the principal water distribution channel of the community source (*n*=16). The water samples will be analysed *in situ* at the schools and households for turbidity, pH and chlorine residual using the *DelAgua* kit (Oxfam-DelAgua; Guildford, UK) using readily available SOPs [54]. If the concentration of free chlorine residual is greater than 0.2 mg/l (0.2 ppm) and the turbidity less than 5 turbidity units, the water samples will not be analysed for TTC [54]. If the results do not meet these criteria, water samples will be transported in cool boxes to the laboratory in Kirnetar Health Centre and stored in a refrigerator at 4 °C before analysis using the *DelAgua* kit. The water samples will be tested for CFU/100 ml according to WHO drinking water standards [53].

Quality control will be conducted with 10 % of all water samples collected by independent laboratories.

### Questionnaire survey with schoolchildren and their caregivers

The KAP survey was established with the guidelines and KAP manual provided by FAO, using standardised questions and amendments by the Swiss TPH research team [55]. Children’s caregivers will also be invited to respond to a questionnaire investigating sociodemographic, -economic, health and food security issues. The questionnaire surveys for children and their caregivers will be tablet-based using the Open Data Kit software [56].

### Focus group discussions and in-depth interviews

Focus group discussions (FGDs) will be conducted with 6 to 10 randomly selected caregivers from sampled children in each school to better understand the caregivers’ perceptions on nutrition and health. The school director and teachers will be interviewed with a semi-structured questionnaire to record characteristics of children’s health challenges, and to discuss children’s nutrition and health education in the curricula, school health activities, school water and sanitary installations and, if existing, the school feeding programmes.

### Data management and analysis

Quantitative data from stool and urine examinations, anthropometrics and Hb measurements will be entered in Microsoft Excel 2010 (Microsoft; Redmond, USA). A double data entry system will be used to ensure data quality. Data will be evaluated for discrepancies and validated after removing inconsistencies. The z-score values for height-, weight- and BMI-for-age relative to the WHO 2007 reference will be calculated using WHO AnthroPlus (WHO; Geneva, Switzerland). Statistical analyses will be carried out with Stata version 13 (StataCorp; College Station, USA). Analysis of baseline data will be conducted to describe the prevalence of malnutrition, intestinal parasitic infections, WASH conditions, KAP and basic socioeconomic characteristics. Logistic regression models will be employed to estimate associations of malnutrition, intestinal parasitic infections and anaemia with risk factors.

FGDs and in-depth interviews (IDIs) will be transcribed, translated into English by bilingual research assistants and entered as Microsoft Word documents into MAXQDA version 11 (VERBI GmbH 2012; Berlin, Germany) for data coding and analysis. Main themes will be identified and coded in order to categorise explanations and descriptions of nutritional and health related perceptions and issues.

Longitudinal analysis will be conducted to evaluate the intervention effects of the complementary interventions under study. The results from the different study arms will be compared at the end of the 1-year intervention period.

### Data storage and handling

All data files will be stored in a secure server at Swiss TPH. ID codes and name-linked information on participants will remain confidential and will be used only to communicate clinical results to participants for their respective treatments.

### Ethical considerations

The two research protocols for Burkina Faso and Nepal were reviewed by (i) the Institutional Research Commission of Swiss TPH (reference number FK 116; date of approval 30 October 2014); (ii) the “Ethikkommission Nordwest- und Zentralschweiz” (EKNZ) in Switzerland for the Nepal study protocol (reference no. UBE-15/02; date of approval 12 January 2015); (iii) the EKNZ in Switzerland for the Burkina Faso study protocol (reference no. 2014–161; date of approval 19 January 2015); (iv) the “Comité d’Ethique pour la Recherche en Santé, Ministère de la Recherche Scientifique et de l’Innovation, et Ministère de la Santé” in Burkina Faso (reference no. 2014-5-058; date of approval 20 May 2014); (v) the “Institutional Review Committee of Kathmandu University School of Medical Sciences, Dhulikhel Hospital”, Nepal (reference no. 86/14; date of approval 24 August 2014); and the (vi) Institutional Review Committee, Health Research Council, Nepal (reference no. 565; date of approval 11 November 2014).

The two studies have been registered under a single trial registration number at the International Standard Randomised Controlled Trial Number Register ISRCTN30840 (date assigned: 17 July 2015; http://www.isrctn.com/ISRCTN17968589).

## Discussion

Malnutrition and intestinal parasitic infections are a major burden on children’s health globally and particularly in LMIC, including Burkina Faso and Nepal. Inadequate WASH conditions play an important role in the high burden of communicable diseases [21, 57]. The morbidity due to malnutrition, intestinal parasitic infections and diarrhoeal diseases in Burkina Faso and Nepal continue to be considerable [4]. Given the global persistence of malnutrition and ill-health, the research and international development communities are increasingly paying attention to enhancing nutrition and health as the primary goals and outcomes of food production and delivery systems [58–60]. Agriculture as a source of nutritious food and well-being has recently been recognised and is addressed in the new Sustainable Development Goals (SDGs), particularly in SDG 2: “End hunger, achieve food security and improved nutrition and promote sustainable agriculture” [61]. There is, however, an insufficient evidence-base which supports these agriculture, nutrition and health linkages [58–60]. Indeed, according to Masset et al. (2011), who undertook to date the largest systematic review on agricultural intervention to improve the nutritional status of children, there is “no evidence of the impact [of agricultural interventions] on prevalence rates of stunting, wasting and underweight among children under five” [62]. Even though agriculture interventions were beneficial in promoting consumption of nutritious foods, evidence of improved nutritional indicators was not consistent [62–64]. However, according to Webb (2013), the lack of evidence on the impact of agricultural interventions on nutrition and health outcomes should not be attributed to the inefficacy of these interventions, but rather to insufficient statistical power (small simple size), lack of rigorous counterfactual analysis, inadequate selection of outcome indicators for the kind of interventions considered, and few accounted for the heterogeneity of impacts even when they were positive [60, 62, 64, 65]. Furthermore, school-aged children are moving into the focus of recent initiatives by governments, bilateral and multilateral organisations, and other development actors; which have recognised the benefits of good health and nutrition of schoolchildren to contribute to educational achievement, growth and development [3, 66–70].

The two studies in Burkina Faso and Nepal within the frame of the overall VgtS project that we describe here will support the reinforcement of this recent attention on schoolchildren’s nutrition and health by focusing on schools as an entry point for health promotion, infection control and life-skills education. Moreover, the studies will contribute to fill existing data gaps on schoolchildren in these two countries, concurrently identifying their nutritional and health challenges and needs. The data collected will serve to inform the design of appropriate and tailored school-based interventions with close participation of the local community, school teachers and directors, as well as with the local research and VgtS project team. The precise set of interventions will be developed after the baseline survey in Burkina Faso and Nepal. The interventions will be designed with a multidisciplinary team of educators, epidemiologist, nutritionist, parasitologists and WASH experts in order to improve water quality, sanitary and hygiene environments and to translate the nutritional and health risk factors into effective educational messages, thereby encouraging schoolchildren to change their behaviour.

The two studies also aim to address the scientific research gap by conducting rigorous intervention studies and quantifying the possible effects of complementary school garden, nutrition and WASH interventions. With the two particular study designs as suggested in Burkina Faso and Nepal, we will be able to analyse the different types of school garden, nutrition and WASH intervention packages. While in Burkina Faso the focus will be on the comparison of integrated and complementary school garden, nutrition and WASH interventions (arm A) as compared to the control schools with no interventions (arm B); in Nepal, we will additionally be able to conduct comparisons between these two study arms to the school garden intervention schools (arm C) and the nutrition and WASH intervention schools (arm D).

Several considerations underscore the relevance for the two concerted and complementary study designs. First, with the same research methods and questionnaire tools applied, data collected from Burkina Faso and Nepal will be used for comparative analysis. Second, the two similar study designs will offer strategies for comparing different public health approaches with an emphasis on schoolchildren’s health and will provide opportunities for discussing the long-term sustainability of these programmes, especially in areas where the targeted diseases are highly prevalent.

Taken together, the overarching goal of the two studies is to assess the potential of suitable interventions to improve health of school-aged children in resource-constrained settings. The insights gained will contribute to estimate the burden of malnutrition and intestinal parasitic infections in schoolchildren and may provide guidance for future research activities, for the implementation of health policies at the school level, as well as for future public health recommendations and health policy planning.
