# Erratum to: Complementary school garden, nutrition, water, sanitation and hygiene interventions to improve children’s nutrition and health status in Burkina Faso and Nepal: a study protocol

**DOI:** 10.1186/s12889-016-3134-6

**Published:** 2016-07-05

**Authors:** Séverine Erismann, Akina Shrestha, Serge Diagbouga, Astrid Knoblauch, Jana Gerold, Ramona Herz, Subodh Sharma, Christian Schindler, Peter Odermatt, Axel Drescher, Ray-yu Yang, Jürg Utzinger, Guéladio Cissé

**Affiliations:** Swiss Tropical and Public Health Institute, P.O. Box, CH-4002, Basel, Switzerland; University of Basel, P.O. Box, CH-4003, Basel, Switzerland; Kathmandu University, P.O. Box 6250, 45200 Dhulikhel, Nepal; Institut de Recherches en Sciences de la Santé, P.O. Box 7192, Ouagadougou, 03 Burkina Faso; University of Freiburg, Friedrichstr. 39, D-79098 Freiburg im Breisgau, Germany; AVRDC - The World Vegetable Center, P.O. Box 42, 74151 Shanhua, Taiwan

## Erratum

After publication of the original article [1], it came to the authors’ attention that the Trial registration URL provided in the abstract was not correct. An internal link was supplied, so the original article has been updated to include the correct URL (http://www.isrctn.com/ISRCTN17968589) in the abstract.
